# Virtual reality or personal computer-based gynecologic pelvic exam simulation: medical student preferences

**DOI:** 10.1186/s12909-025-06757-z

**Published:** 2025-02-24

**Authors:** Rosalyn E. Plotzker, Derek J. Harmon, Tim Kanellitsas, Barbie A. Klein

**Affiliations:** 1 Department of Epidemiology and Biostatistics, University of California, San Francisco (UCSF), 550 16thStreet, Mission Hall, Box 0560, San Francisco, CA 94143 USA; 2 The Ohio State University College of Medicine, Division of Anatomy, 440 Hamilton Hall, 1645 Neil Ave, Columbus, OH 43210 USA; 3 Kairos XR, 351A Frederick Street, San Francisco, CA 94117 USA; 4 University of California, San Francisco (UCSF), Department of Anatomy, School of Medicine and the Department of Cell and Tissue Biology, School of Dentistry, 513 Parnassus Ave, Room 1320D, Box 0452, San Francisco, CA 94143 USA

**Keywords:** Virtual reality, Simulation, Pelvic exam, Medical education

## Abstract

**Background:**

Gynecologic pelvic exams (GPEs) are a required proficiency for healthcare trainees, yet practice opportunities are limited. The Virtual Approach to Gynecology Project aims to supplement traditional learning with a virtual-based GPE module. This study compares trainee experiences using the immersive virtual reality (VR) version to an equivalent non-immersive personal computer (PC) simulation.

**Methods:**

Five groups of 3–5 preclinical medical students were randomized to complete one version of the GPE simulation (either the PC or VR), followed by written feedback and a structured focus group. Each group then completed the other version, after which a second written feedback form was completed, and a final focus group conducted. Focus group comments were recorded, transcribed verbatim and coded. Thematic analysis was performed on coded comments and analysis of written feedback compared Likert-scale responses of VR and PC versions.

**Results:**

Twenty-two individual students enrolled and were assigned to one of five groups. Focus group discussions yielded a total of 138 responses that underwent thematic analysis. VR was unanimously preferred to the PC version, scoring significantly higher Likert-scale responses on ease of use, realism, improved comfort, and confidence. The following 3 themes emerged from the thematic analysis: (1) realism and immersiveness, (2) ease of use, and (3) enjoyment. Compared to PC, VR was found to be more realistic and enjoyable, though both versions had some reported difficulty regarding use.

**Conclusion:**

The immersive VR-based simulation provided a more realistic and enjoyable experience for the GPE simulation compared to the PC-based simulation and was unanimously preferred.

**Supplementary Information:**

The online version contains supplementary material available at 10.1186/s12909-025-06757-z.

## Background

Proficiency with the gynecologic pelvic exam (GPE) is a fundamental skill in primary care that is initially learned during undergraduate medical training. Yet evidence suggests approximately half of primary care providers do not consistently perform GPEs [1]. Among providers, common barriers to performing a GPE include lack of knowledge, skill, and/or experience, which point toward deficiencies in training and opportunities to practice the exam [2–4]. For early trainees, opportunities to observe the GPE are limited by availability of a clinical gynecologic setting, preceptorship, and patient willingness. In addition, early healthcare trainees often experience anxiety when first conducting breast and anogenital exams including GPE on live patients, which pose a further barrier to learning [5]. Pelvic mannequins and 2-dimensional videos of the GPE are simulation modalities commonly employed to prepare trainees for live person experiences with standardized patients (SPs) and with true patients on clerkship rotations [6, 7]. While both have shown benefits compared to no simulation, these approaches may be suboptimal considering their lack of realism, fidelity and immersion [8–10]. An additional learning step to span this gap between non-immersive simulation and live-person experience, therefore, may be to incorporate an immersive simulation curriculum.

Immersive virtual reality (VR) technology has shown promise in medical education as a strategy to overcome barriers associated with teaching skills that are difficult to acquire in clinical training, although empirical evidence demonstrating the advantage of VR over desktop computer simulations has been limited thus far [11]. The Virtual Approach to Gynecology Project was created to overcome barriers to GPE learning in early medical education with the creation of a 360-degree video depicting a GPE and the subsequent development of a software simulation designed for both a non-immersive personal computer (desktop or laptop, herein referred to simply as “PC”) and VR. This study aimed to assess the perceptions and preferences of the VR compared to PC versions of the GPE modules among first and second year pre-clinical medical students.

## Methods

The study was reviewed by the University of California, San Francisco Institutional Review Board and determined exempt (Protocol #21–33982). Informed consent to participate was obtained from all participants in the study.

### Application design and development

#### Overview of application curriculum

**Fig. 1:**
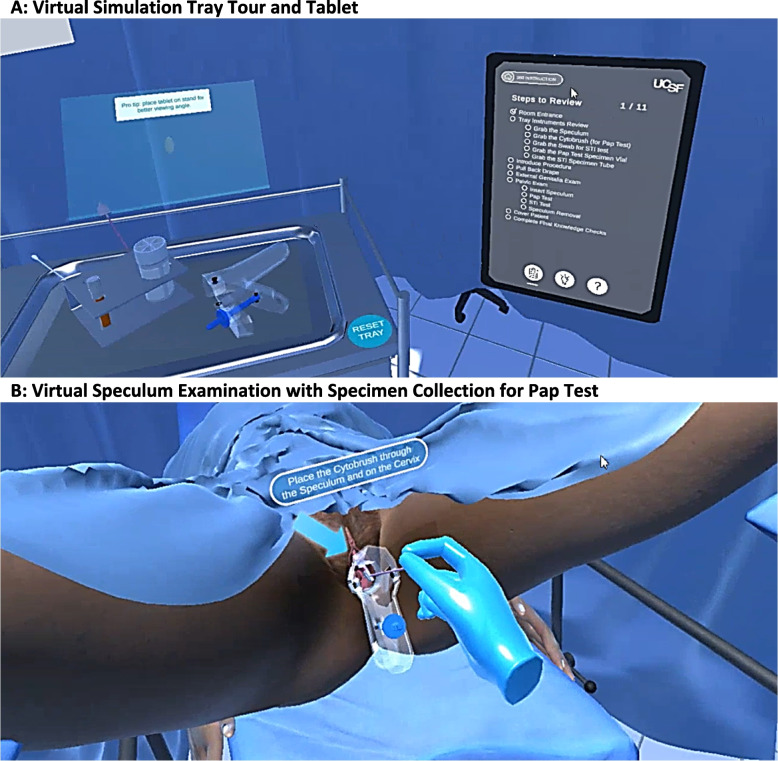
The Virtual Approach to Gynecology, Selected Images from Modules 2 and 3. **A** Virtual Simulation Tray Tour and Tablet. **B** Virtual Speculum Examination with Specimen Collection for Pap Test. The user is instructed to perform specific actions: locking the speculum into place, inserting and rotating a cervical broom multiple times for the cervical cytology collection, inserting the STI swab and sampling both sides of the vaginal wall, placing specimens in the appropriate vials, then unlocking and removing the speculum, and finally re-draping the patient

The Virtual Approach to Gynecology Project application consists of three modules. In module 1, a 360-degree video of a GPE is shown from the provider viewpoint. In module 2, the user enters a virtual space where they participate in a guided interactive simulation of a GPE. As a part of the simulation, the participants review the examination instruments (e.g. gynecologic speculum, cervical cytology “Pap test” and sexually transmitted infection (STI) swab materials), and perform the procedure steps modeled directly from the 360-degree video in a guided format. (Fig. 1A and B). In module 3, the user performs the same simulation conducted in module 2, but without the use of the guide. Throughout modules 2 and 3, a virtual tablet serves as an interface for the user and tracks the user’s progress. Short multiple-choice question knowledge checks are provided via the tablet between modules to assess learner comprehension for module content (e.g. clinical skills, anatomy knowledge). The following sections provide details of each module.

#### Module 1: 360-degree video

A GPE performed by a licensed obstetrics and gynecology clinician (“provider”) with a “patient” (portrayed by a gynecologic teaching assistant/standardized patient) was recorded with a 360-degree video camera. The camera was positioned between the provider and patient to give the viewer the provider’s perspective. The video depicts the provider asking the patient’s permission to enter the exam space to ensure patient readiness, lifting the drape, examining the vulva, inserting the speculum and visualizing the cervix, and collecting specimens for routine screening (a cervical cytology “Pap test” with cervical broom and vaginal swab for gonorrhea and chlamydia screening). This 360-degree video was used as the basis for the VR and PC software applications.

#### Modules 2 and 3: Interactive software

The interactive software application for this study was developed for two different hardware types: VR Oculus Quest 2 headsets (Meta Platforms Inc., Menlo Park, CA) and PC (i.e., desktop or laptops). While each hardware differed in user experience and degree of interaction, the structure, sequence, and user interface were identical across both mediums. The interactive applications for both the PC and VR simulation were built using the Unity 3D Engines (Unity Technologies, San Francisco, CA), enabling the repurposing of 80% of the architecture for both platforms.

Module 2 begins by introducing the user to the environment, the virtual tablet, and their virtual tutor – a virtual avatar named “Uty" provides verbal instructions which are also written in adjacent text boxes. The user is instructed on how to request permission to enter the exam room. Once inside the examination space, an introductory “tray tour” familiarizes them with the instruments (Fig. 1A). In VR, hand-held controllers are used to manipulate the instruments, while the computer version utilizes a mouse and keyboard. After the tray tour, the user is instructed to ask for consent from the patient to lift the drape. Once the patient is undraped, vulvar anatomy is reviewed with supplemental anatomical diagrams alongside the virtual patient. Next the user is guided through the speculum exam. The user is instructed to perform specific actions: locking the speculum into place, inserting and rotating a cervical broom multiple times for the cervical cytology collection, inserting the STI swab and sampling both sides of the vaginal wall, placing specimens in the appropriate vials, then unlocking and removing the speculum, and finally re-draping the patient (Fig. 1B). In module 3, the virtual tutor and introductory tray tour are absent. The user is expected to perform each step sequentially without prompts.

### Participants and setting

This study took place at a large urban academic medical institution. All first- and second-year medical students at the principal investigator’s institution were recruited to participate in this study via email, student online forum post, and an announcement made before a gross anatomy lecture. As an incentive to participate in the study, students were offered to receive a $20 gift card. Students who responded with an interest in participating were scheduled into one of five group sessions based on availability, with a maximum of five students per group. Focus groups were conducted between November 2021 and April 2022, and did not correspond to gynecologic-related learning blocks within the wider medical school curriculum. First year students had yet to complete the endocrine and reproductive learning blocks, while second year students had completed those blocks.

### Study design and procedures

**Fig. 2:**
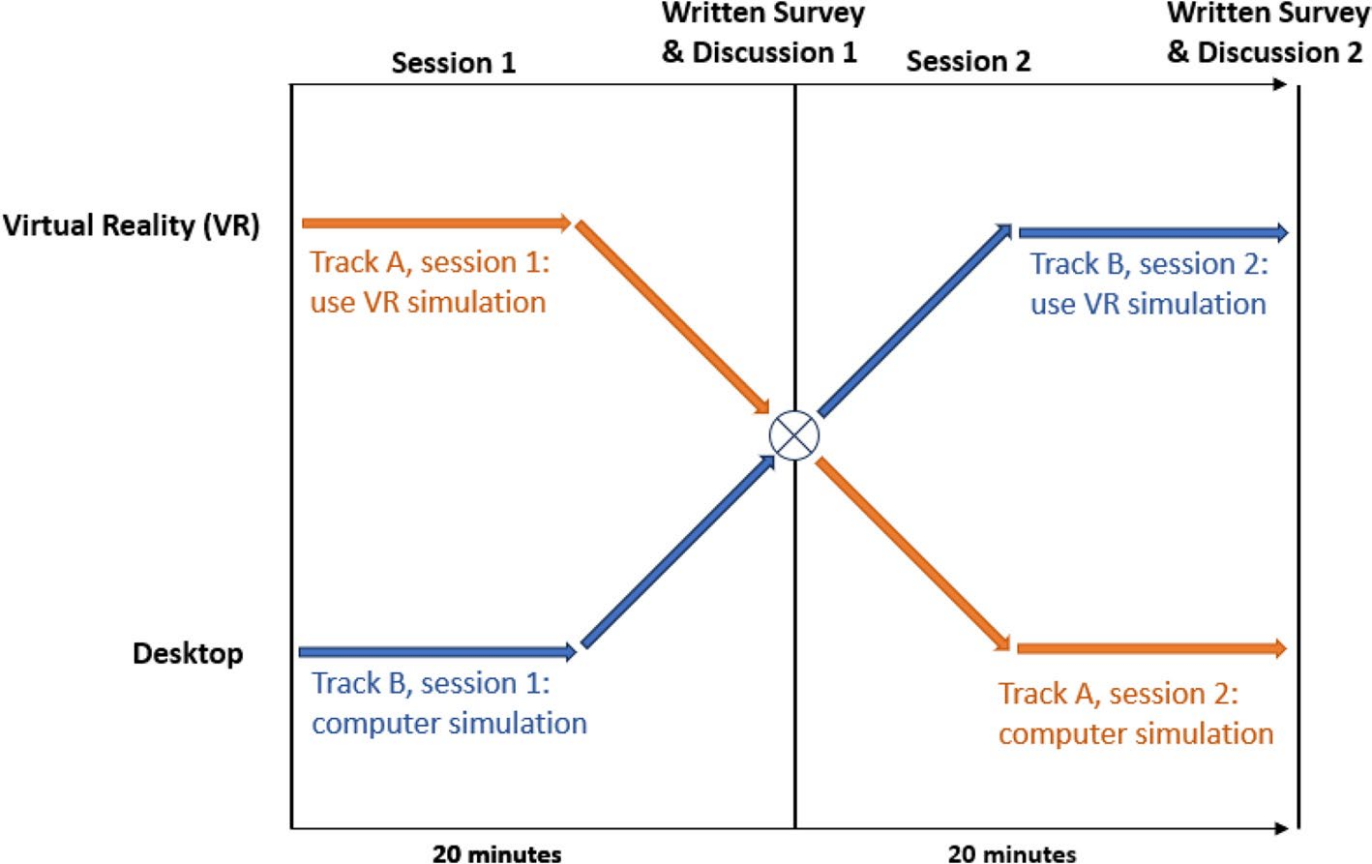
Two by two crossover study design

This study utilized a randomized 2 × 2 crossover design (Fig. 2). After the groups were assigned to a scheduled date, the authors randomly assigned each group to begin with either the VR or PC version of the simulation. Due to the first-time use of VR headsets for many participants, study staff were available for participant questions related to VR headset use. Twenty minutes were allotted for the first simulation session, followed by time for written feedback, and then focus group discussion. After the first focus group discussion, participants immediately began a second twenty-minute simulation session using the other device (e.g. if starting with PC, they would follow with VR). Once completed, participants answered a second set of written questions about the second simulation experience, as well as questions comparing PC to VR versions of the application, and questions about their attitudes toward VR in medical education generally. Finally, when all participants completed their forms, a final focus group was conducted. Both focus group sessions for a scheduled group were recorded and then transcribed verbatim by one author (RP).

### Outcome instruments

Written surveys and focus group discussion prompts were adapted from previously validated instruments that were used for an internal evaluation of the 360-degree video module alone (see Supplement). The internal evaluation was conducted in 2020, however it was discontinued due to COVID-19 restrictions.

#### Written survey

Participants were first asked to rate their familiarity with VR (“Not Familiar”, “Rarely Use”, “Somewhat Familiar”, and “Very Familiar”). The survey then included a 4-point Likert-scale (“Disagree”, “Somewhat Agree”, “Agree”, and “Strongly Agree”) for the following statements referring to the simulation:

- This was easy to use
- This felt like a realistic experience
- This could help me feel more comfortable performing pelvic exams on live patients
- This could help me feel more confident performing pelvic exams on live patients
- I would recommend this to a colleague

Likert-scale responses were converted to numeric scores 1–4, where 1 corresponded to “Disagree” and 4 corresponded to “Strongly Agree”. Scores for each of the five metrics were then averaged for the PC and VR responses and compared. Statistical significance was tested with Wilcoxon Rank Sum.

#### Focus group interviews

The first focus group discussions were prompted by the following open-ended questions, which were asked sequentially by the facilitator, an assistant professor in the epidemiology and biostatistics department who had no previous relationship to the participants (RP):

- (1) What are your thoughts about this application?
- (2) What were your impressions, or emotional responses while you were using the application?
- (3) How could you envision using this module in the future?

The second focus group discussion consisted of the following prompts:

- (1) What are your thoughts about the VR and the desktop (i.e. PC) in comparison?
- (2) Do you prefer the VR or desktop version and why?
- (3) How do you envision using this module in the future, not just individually, but for education in general? Where would this fit in in your education?

### Statistical analysis

Likert-scale analysis compared scoring for VR and PC modalities for all five metrics. Wilcoxen Rank Sum was used to determine statistically significant differences.

Focus group transcripts and open-ended narrative data from the focus group guide were subjected to open coding using Microsoft Excel (Microsoft Corp., Redmond, WA) by two authors (RP and BK). Individual comments from focus group participants were coded. Codes were developed directly from the data, were not predetermined, and then were condensed into parent codes. Consensus of coding interpretation was reached, and coding discrepancies were reconciled between two authors (RP and BK).

## Results

Of 162 first-year and 172 second-year eligible medical students, a total of 22 participants enrolled in the study and were assigned to one of five groups, with 3 to 5 participants per group. Per both written questionnaire and focus group discussions, students unanimously expressed a preference for the VR simulation over the PC simulation after both modalities were completed, regardless of which simulation the students completed first.

### Written assessment results

Ten (45%) participants reported they rarely used VR, 9 (41%) were not familiar with VR at all, and the remaining 3 (14%) were somewhat familiar with VR.

Compared to the PC version, participants scored the VR experience significantly higher for all 5 metrics (Table 1), regardless of the order in which the modalities were used. The average scores for ease of use for VR and PC were 2.5 and 1.9, respectively (*p* = 0.03). Average scores for realism for VR and PC were 3.0 and 1.9, respectively (*p* < 0.001). Average scores for improving comfort with a GPE for VR and PC were 3.5 and 2.7, respectively (*p* < 0.001), while improving confidence were 3.6 and 2.5 for VR and PC, respectively, (*p* < 0.001); finally, when asked “would you recommend this to a colleague?” average scores were 3.5 and 2.4 (*p* < 0.001) for VR and PC versions, respectively.

**Table 1:** Likert-scale scoring for Virtual Reality (VR) and personal computer (PC) modalities (*n*-22)

Question	VR average	PC average	*P*-value
“Was it easy to use?”	2.5	1.9	0.03
“Was it realistic?”	3.0	1.9	< 0.001
“Did it improve your comfort?”	3.5	2.7	< 0.001
“Did it improve your confidence?”	3.6	2.5	< 0.001
“Would you recommend it to a colleague?”	3.5	2.4	< 0.001

### Focus group discussion

During focus groups, every student participant responded to each question prompt posed to the group. A total of 138 individual comments, which were evenly distributed across focus groups and participants, underwent qualitative content analysis, were coded, and further categorized into parent codes. Of these, 72 (52%) pertained to the PC version while 66 (48%) pertained to VR. Three main themes of facilitators/barriers to learning emerged through transcript qualitative analysis: 1) realism/immersiveness (*n* = 57); 2) ease-of-use (*n* = 28); and 3) enjoyment (*n* = 16).

**Fig. 3:**
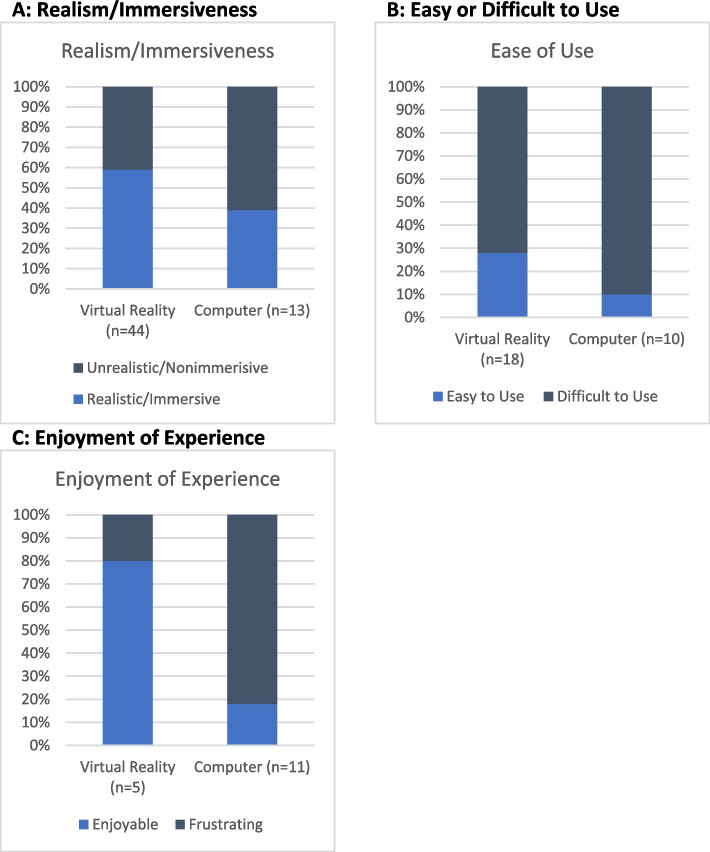
Focus Group thematic analysis (Total Comments: *N* = 138). **A**: Realism/Immersiveness. **B**: Easy or difficult to use. **C**: Enjoyment of experience

The VR version was found to be more realistic and immersive. (Fig. 3A) Realism/immersiveness was the most widely commented aspect for the headset-based application, generating 44 comments. The majority (59%) of comments affirmed it was realistic/immersive, specifically noting the importance of movement and spatial orientation:

“Compared to the desktop version you really have to be physical and so at one point I tried to use my right hand to open the labia and then I realized I wasn’t able to move forward so I had to use my left hand and that was really good because I think it helped me think more spatially about how I would have to do the exam.” (Group 4 participant #1).

Another student added:

“It was very cool how everything is 3D and you can move around. It feels like you’re practicing picking up the speculum and actually doing it. I think if you did that before an SP [standardized patient] you would be super prepared. Pulling the drape and doing all of that for the first time when you’re with an SP for the first time, it’s all kind of scary” (Group 4 participant #2).

Interaction in combination with physicality with the VR version was also appreciated, as one participant noted, “the VR was much better than the desktop version because you could move around and see the tools properly and interact with everything; overall, it’s just a much better simulation of what the experience is like” (Group 5 participant #1).

Comparatively, PC discussions yielded 13 comments pertaining to realism/immersiveness, however most (61%) noted a lack of realism: “the computer was so difficult because nothing felt intuitive” (Group 1 participant).

Comments on ease-of-use arose for both VR (*n* = 18) and PC (*n* = 10); most comments identified difficulty. (Fig. 3B). For the computer experience, 90% of comments noted the app was difficult to use, compared to 72% for the VR version.

Finally, comments revealed that, overall, users said the VR version was enjoyable or fun, while they reported the PC version was frustrating and cumbersome. (Fig. 3C). Of the 11 comments related to enjoyment, 82% complained the PC version was tedious. In comparison, although only 5 comments reflected on the enjoyability of the VR-based experience, 4 (80%) found it to be a fun experience: “It was super exciting to kind of be there and I got very lost in it. Very easy to like lose track of time. I was like, ‘Wow it’s only been five minutes they’re saying it’s been 20’ and it made me excited to learn” (Group 3 participant #1).

In addition to the above themes, a few participants reflected on the application’s impact on their comfort and confidence related to performing a GPE, as well as the use of VR in medical education more generally. Consistent with the survey data, several trainees attested to VR improving both their comfort and confidence in performing a gynecologic speculum exam. Interestingly, some students noted that the VR-based versions made them more considerate of the virtual patient’s experience:

“I found myself being very aware of making sure that I didn’t like scoot up too fast on the patient. Even though it’s, you know, fake I was very conscious of moving my hand and practicing all of the steps… Just making the patient feel more comfortable… there [were] vibrations with the handsets… it was a good reminder of not being so aggressive. I just felt like there was a lot more patient-centered things that I was thinking about with the VR.” (Group 5 participant #2).

Finally, several participants envisioned VR could be used as a preparatory simulation prior to working with standardized patients: “I felt like I knew more about how to do a Pap smear than after our actual current medical education when they taught us. I feel like in our real medical education we had the actual SPs for the speculum exam that was obviously – inserting speculum is very unique and something that you need with patients, but for like understanding the process and what things look like and all that I thought it [the VR experience] was fantastic.” (Group 3 participant #2).

## Discussion

We created an immersive VR curriculum that shows promise to fill a potential gap in GPE education. To our knowledge, this was the first study to develop and compare identical PC-based and VR-based simulation software for a GPE. Ease of use, realism, improving comfort, improving confidence, and recommended use were all scored significantly higher for the VR-based simulation via written feedback, consistent with earlier studies evaluating VR acceptability in medical education [12–14]. In addition, written response and focus group interviews revealed that, when compared to an identical PC simulation, VR was unanimously preferred. This preference existed regardless of one’s prior VR experience and the order in which the modalities were used.

A potential drawback of VR-based learning is user inexperience with the technology compared to the ubiquitous use of laptops and desktop computers [15, 16]. Specific issues relating to ease versus difficulty-of-use for VR headsets highlight that deliberate in-app training would be beneficial to orient users, particularly those who are less familiar with using VR headsets. As VR, augmented reality and extended reality emerge in education and in the mainstream, we anticipate users will become more familiar with this technology, and thus this constraint will become less problematic. Interestingly, the PC version of the simulation was also noted to have difficulties, indicating that some of the problems related to user-friendliness was due to the application itself, rather than modality.

Consistent with prior studies exploring VR usefulness, focus group participants frequently highlighted the importance of immersiveness and realism [15, 17, 18]. Participants often noted the value of physically turning their body or reaching for instruments, emphasizing the value of embodied cognition, even demonstrating the physical movements as they spoke during their discussion [19]. While early medical education is dominated by cognitive learning that relies on factual recall and intellectual reasoning, gaining proficiency in physical exam skills requires the added component of simulation and motor learning, ideally inclusive of movement and familiarity with the surrounding environment (e.g. location of the tray, orientation to the patient) [20–22]. Thus, immersive learning – in which a user physically experiences the procedure – may reinforce cognitive learning and also address the embodied aspects of the exam [19]. VR offers a responsive platform to integrate elements of gamification such as: appropriate increase in challenge (i.e., game-like environment criterion); active engagement with minimum guidance (i.e., learning-by-doing criterion); and ability to teach academic content in an engaging format to increase learner motivation (i.e., instructional objectives criterion). The use of VR and gamification in education, broadly, has been shown to improve learning, motivation to participate, reported self-efficacy and learning satisfaction; in addition student engagement in the forms of curiosity, attention, and imagination are positively impacted through VR gamification [23].

Regarding comfort and confidence, all users felt that this simulation improved their sense of self-efficacy in performing a GPE, which was more prominent for the VR simulation than the PC simulation, potentially due to immersive and realistic ratings associated with the VR. However, it was commonly stated that simulation – whether through VR or PC – would not replace actual interactions with patients. One shortcoming of most free-standing virtual rehearsal is the inability to palpate, or gain dexterity needed to easily identify the cervix (e.g. in the setting of a very anteverted or retroverted uterus). Specialized VR programs can be designed that use accompanying models (such as a pelvic model); however, these approaches have the limitation of high cost, and require access to the models themselves rather than an application that can be used with any VR headset in any location [24].

### Study limitations

Our study has several limitations. First, this work included a small sample size of 22 pre-clinical medical students. It also took place at one university, and therefore, may not be generalizable to other institutions or other pre-clinical healthcare trainees. In addition, our application was designed for right-handed individuals, and the application was not assessed for users who were required to use their non-dominant hand, which could prove difficult in a real-world setting. Regarding user difficulty versus ease-of-use, because both versions had reported difficulty, we were unable to distinguish what difficulties were due to the hardware type versus the app itself. With regard to focus group conversations, participants often expressed agreement with other group members. While this may suggest groupthink, we found similar agreement among individual written feedback that was submitted prior to group discussions, suggesting the consensus was not solely groupthink. Finally, research staff were available to assist participants if there was a major technical difficulty, which may not be the case if users were independently using the application outside of a research setting.

### Future directions

VR shows promise for education around learning sensitive exams such as urogenital procedures and may be further developed to include complex or rare pathologies. Thus far, VR has successfully been incorporated into multiple realms of medical education, including surgical training, ultrasound training, airway management training, and the establishment of competencies, such as Entrustable Professional Activity [25–29]. Of note, VR cannot be a stand-alone curriculum. Because VR is a non-tactile rehearsal space for learning steps within a simulated environment, it does not include tactile aspects such as palpation. Thus, VR curricula should be accompanied by additional learning approaches that include physical models and/or standardized patients to simulate aspects of procedures that involve palpation, and the use of real-world instruments. As discussed by students, this immersive modality may serve as an important bridge between non-immersive simulations with mannequins and learning with live patients. Therefore, implementing this application via a stepwise simulation-to-live patient learning approach, may be most impactful. Finally, formal implementation of this application can drive further scholarship on VR acceptability among healthcare trainees.

## Conclusion

The Virtual Approach to Gynecology Project’s VR simulation provided users with an immersive learning experience for sensitive exam rehearsal that was rated superior to a PC version. As VR, and more broadly extended reality, continue to expand in medical education, these technologies can be leveraged to meet learner needs particularly for the development of clinical skills for which practice opportunities in real-life are limited.

## Supplementary Information

Supplementary Material 1.

Supplementary Material 2.

Supplementary Material 3.

## Data Availability

The datasets used and/or analysed during the current study are available from the corresponding author on reasonable request.

## Abbreviations

GPE: Gynecologic Pelvic Exam
PC: Personal Computer
STI: Sexually Transmitted Infection
UCSF: University of California, San Francisco
VR: Virtual Reality
