# Supplementary material for: Virtual reality or personal computer-based gynecologic pelvic exam simulation: medical student preferences
Source: BMC Med Educ. 2025 Feb 24;25:294. doi: 10.1186/s12909-025-06757-z (PMC11849286; doi:10.1186/s12909-025-06757-z)
Supplement: Supplementary file 1 — Supplementary Material 1. [file 12909_2025_6757_MOESM1_ESM.docx]

**Focus group discussion questions after first application use**

1. What are your initial thoughts about this application?
2. What was your impression of this application?
3. How would you envision this type of learning being incorporated into medical education in the future?

**Focus Group discussion questions after second application use**

1. What are your thoughts about the VR versus the desktop [PC] version?
2. Do you have a preference between the two versions?
3. How would you envision this second type of learning being incorporated into medical education in the future?
