# Supplementary material for: Virtual reality or personal computer-based gynecologic pelvic exam simulation: medical student preferences
Source: BMC Med Educ. 2025 Feb 24;25:294. doi: 10.1186/s12909-025-06757-z (PMC11849286; doi:10.1186/s12909-025-06757-z)
Supplement: Supplementary file 2 — Supplementary Material 2. [file 12909_2025_6757_MOESM2_ESM.docx]

**First Evaluation**

*Your feedback is extremely valuable to us in our efforts to evaluate this immersive, interactive virtual training module designed to demonstrate how to perform a routine pelvic exam.*

Please circle the version you used to complete the virtual simulation:

**Virtual Reality (VR) version** – or –               **Desktop version**

Please answer the following questions based on your initial impressions of this application           (place an X in the appropriate box):

|  | Disagree | Somewhat Agree | Agree | Strongly Agree |
| --- | --- | --- | --- | --- |
| This was easy to use |  |  |  |  |
| This felt like a realistic experience |  |  |  |  |
| This could help me feel more comfortable performing pelvic exams on live patients |  |  |  |  |
| This could help me feel more confident performing pelvic exams on live patients |  |  |  |  |
| I would recommend this to a colleague |  |  |  |  |

1. What did you find most beneficial about this application?
2. What did you find most beneficial about this version of the application?
3. What did you like least about this version of the application?

*Thank you for helping to improve medical education!*
