# Supplementary material for: Virtual reality or personal computer-based gynecologic pelvic exam simulation: medical student preferences
Source: BMC Med Educ. 2025 Feb 24;25:294. doi: 10.1186/s12909-025-06757-z (PMC11849286; doi:10.1186/s12909-025-06757-z)
Supplement: Supplementary file 3 — Supplementary Material 3. [file 12909_2025_6757_MOESM3_ESM.docx]

Please circle the version you used to complete the virtual simulation:

**Virtual Reality (VR) version** – or –               **Desktop version**

Please answer the following questions based on your initial impressions of this application           (place an X in the appropriate box):

|  | Disagree | Somewhat Agree | Agree | Strongly Agree |
| --- | --- | --- | --- | --- |
| This was easy to use |  |  |  |  |
| This felt like a realistic experience |  |  |  |  |
| This could help me feel more comfortable performing pelvic exams on live patients |  |  |  |  |
| This could help me feel more confident performing pelvic exams on live patients |  |  |  |  |
| I would recommend this to a colleague |  |  |  |  |

Which version of this application do you prefer?      (circle one):

**VR version**             – or –               **Desktop version**

1. What did you find most beneficial about this version compared to the other?
2. What did you like least about this version compared to the other?
3. How familiar are you with VR 360-degree video? (select one)
   1. Very – I use VR 360-degree video on a regularly
   2. Somewhat – I have used it in the past occasionally, but do not use it regularly
   3. Rarely use – I’ve had tried it once or twice, but it does not feel familiar to me
   4. Not Familiar – This is my first experience viewing a VR 360-degree video
4. What are your general feelings about using technology like VR and 360-degree video for learning about patient physical exams?
5. What other comments do you have about this application in general and the two different versions (VR and desktop)?
